# Nutritional status of preschool children attending kindergartens in Kosovo

**DOI:** 10.1186/s41043-017-0105-1

**Published:** 2017-06-02

**Authors:** Agim Rysha, Tahire M. Gjergji, Angelika Ploeger

**Affiliations:** 1 Department of Food Technology, Faculty of Agribusiness, University of Pejë, Rr. “UÇK-së”, 30000 Pejë, Kosovo; 2 Clinical Center of Kosovo, Medical Faculty, University of Prishtina, Mather Theresa, NN 10000 Prishtinë, Kosovo; 3 Department of Organic Food Quality and Food Culture, Faculty of Organic Agricultural Sciences, University of Kassel, Nordbahnhofstr. 1a, 37213 Witzenhausen, Germany

**Keywords:** Kosovo, Preschool children, Child underweight, Child overweight

## Abstract

**Background:**

There is very limited data on malnutrition of preschool children in Kosovo. The main objective of the study is to provide a nutritional status profile of preschool children attending kindergartens in Kosovo.

**Methods:**

Cross-sectional study of children aged 12–59 months (*n* = 352 children) and children aged 60–83 months (*n* = 134) enrolled in public and private kindergartens of Kosovo. Anthropometric measurements used for this study are *weight* and *height* of the preschoolers (12–83 months). A measuring board was used for measuring the length/height of children younger than 2 years, while digital weight and height scale *Seca 763* was used for measuring of preschool children taller than 110 and *Seca 213* was used for measuring the height for children who were shorter than 110 cm. Statistical analyses of underweight and overweight trends across sex and age groups as well as between children from public and private kindergartens were carried out. Qualitative variables were tested with a *chi-square* test. The differences between groups were assessed with a *Student t test* for normally distributed variables and a *Mann-Whitney test* for abnormally distributed numerical variables.

**Results:**

The mean z-scores for *weight-for-age*, *height-for-age*, *weight-for-height*, and *BMI-for-age* largely fell within 0.0 and 1.0. The percentage of stunted children is 3%, whereas child wasting is 1.9%. The overall percentage of obese children is 2.3%; furthermore, 8.9% are overweight and 27.3% have a possible risk of being overweight.

**Conclusions:**

The incidence of children underweight is slightly decreasing. The prevalence of overweight and obese children in sample chosen is evident.

## Background

Countries throughout the world are at various stages of nutritional transition and, increasingly, they are documenting that food insecurity and under-nutrition exist side by side with problems of over-nutrition, obesity and chronic diseases [1]. Different diseases of adults are considered to have a close relationship with malnutrition and incorrect or improper nutrition in childhood. The association between childhood diet and being underweight or overweight as children or even as adults has been shown in numerous studies [2–4]. Global evidence indicates increase of obesity, diabetes, cardiovascular diseases, and other chronic non-communicable diseases, especially in developing and transitional countries. This evidence also indicates that disease processes begin early in life, and it is expected that the epidemic will continue to increase due to a lifetime of exposure to poor diet and the influence of different factors [5, 6].

Proper nutrition in childhood is considered to play a crucial role in the physical, mental, and emotional development of children through to their later adult age. Children are therefore considered the priority population for intervention strategies [7]. Nutritional assessment in children is needed to determine their nutritional status and problems in their food regimes and if identified, to treat such problems in order to prevent them from becoming larger and threatening to children’s health. Therefore, many studies are taking place, for example, in preschool institutions and kindergartens.

Anthropometry is a practical and immediately applicable technique for assessing children’s development patterns. Anthropometric indicators can be used as a screening device to identify individuals at risk of under-nutrition or over-nutrition, followed by a more elaborate investigation using other techniques [8]. Complementary to this assessment the *BMI* (*body mass index*) z-scores has been recommended as the most appropriate single indicator of overweight status and obesity in children and adolescents outside of research settings [9–11].

Kosovo, as the youngest country in accession discussion with the EU, is among a few countries without any National Nutrition Strategy or Nutrition Education Program. According to the 2009 Kosovo education statistics, the number of preschool establishments (kindergartens) was 40 with a total of 5091 children enrolled, divided into two groups (a) toddlers aged 1–3 years and (b) preschoolers aged 4–6 years [12]. The percentage of preschool-aged children attending kindergartens in Kosovo is very low (less than 10%). There is a high demand by families to send their children to kindergartens, but a lack of kindergartens and budget limitations prevent many children from attending such preschool institutions.

Based on literature exploration, the statistics and investigation on nutrition and health characteristics of the country seem to be scarce, and there is very limited data on malnutrition and overweight preschool children in Kosovo. According to the survey conducted by UNICEF in July 1999 [13], the acute malnutrition was detected in 3.1% of the children from 0–5 years (including 1% severe) and chronic malnutrition was present in 10.7% of the children (including 3% severe). In 2001, the United Nations Children’s Fund [14], in collaboration with the Institute for Public Health of Kosovo, promoted a survey on the health and nutrition of women and children. Low *height-for-age* was found in 10% of the children aged 6–59 months while the prevalence of low *weight-for-height* was 4%.

The main objective of this study is to provide preliminary data on nutritional status in preschool children attending public and private kindergartens in Kosovo. Since Kosovo did not yet develop its own national standards for nutritional guidelines and nutritional assessment, the growth of preschool children was examined to determine whether and how the anthropometric indicators of selected samples differ from actual WHO standards and references, which are mainly used for the screening, surveillance, and monitoring of preschool children. It is expected that the growth of preschool children in Kosovo is not in line with international standards and references for *weight-for-age*, height-for-age, weight-for-height, and *BMI-for-age.*

## Methods

This cross-sectional study covered four public and one private kindergarten, randomly selected from different regions of Kosovo (Prishtinë; Ferizaj; Kamenicë and Obiliq). The study population included 486 children and the distribution of the study subjects by age and sex is presented in Table 1. Kindergartens in Kosovo are frequented by children of families with different economic, social, and cultural levels.

**Table 1:** Distribution of study subjects by age and sex

Age groups (months)	Gender				Total
	Female		Male		
	*N*	%	*N*	%	*N*
12–23	17	7.7	27	10.2	44
24–35	35	15.8	35	13.3	70
36–47	66	29.7	58	22.0	124
48–59	43	19.4	71	26.9	114
60–71	51	23.0	50	18.9	101
72–83	10	4.5	23	8.7	33
Total	222	100.0	264	100.0	486

All registered children from the selected kindergartens were eligible to participate in the anthropometric assessment. The inclusion criteria for the recruited subjects were (a) children registered and attending the selected kindergarten, and (b) children between 12 and 83 months of age.

In order to conduct a study involving preschool children, institutional consent for access to the kindergartens as well as parental consent was needed. The request for access to preschool settings was addressed to the Ministry of Education, Science and Technology of Kosovo. The Ministry has issued a consent letter, inviting Municipal Education Directors as well as the Kindergarten Directors to support this study. The concept and the objective of the study were explained to the parents and teachers (nursery governess) through group meetings as well as through distributing of an information letter to each parent personally. All parents agreed to have their children participate. An information poster about the start and time frame of the study was placed on the entrance door of each kindergarten prior to the beginning of the research. Anthropometric measurements used for this study are *weight* and *height* of the preschoolers (12–83 months) according to the techniques which were suggested by the WHO [15]. A measuring board was used for measuring the length/height of children younger than 2 years. Digital weight and height scale *Seca 763* was used for measuring the weight of all assessed children and the height of preschool children taller than 110 cm. The measuring range of the equipment is between 110–200 cm with the graduation weight of 50 g and graduation length of 1 mm. With *Seca 763*, both weight and height can be done in one step, calculating the BMI of the patient in an automatic way. Indicated values provide information on the general nutritional condition of the user on the basis of recognized WHO standards*.* Height measuring instrument *Seca 213* with graduation length of 1 mm was used for measuring the height of children who were shorter than 110 cm. This stadiometer is reliable and can be used anywhere because the large floor plate provides the necessary stability, and it is easy to read the results while measuring, thus guaranteeing precise results. The length and height measurement in this study is taken to the nearest 0.5 cm (5 mm). The body weight was measured with the child in underwear or in light clothing, without shoes. The weight measurement in this study is taken to the nearest 0.1 kg. Measurements on weight and height were taken from children aged 12–83 months directly in five kindergartens. The data was recorded by a principal investigator who was supported by trained nurses in conducting the measurement of a child’s length/height and weight. The principal investigator stayed in the kindergartens for the duration of survey. Results of the measurements of weight and height of preschool children in Kosovo have been compared with the growth charts from the World Health Organization. The 2006 WHO Child Growth Standards were applied for the children aged 12–59 months and the Growth Reference 2007 for children aged 60–83 months. Anthropometric indices were constructed by comparing the study data (12–83 months aged children) with those of comparable individuals in the *WHO* reference data. Z-scores (standard deviation score) were used for expressing these comparisons. Anthropometric indicators *WAZ* (weight-for-age z-scores), *HAZ* (*length-or height-for-age z-scores*), *WHZ* (*weight-for-height z-scores*), and BAZ (BMI-for-age z-scores) were used for children of the age group 12–59 months, while weight-for-age (z-scores), height-for-age (z-scores), and BMI-for-age (z-scores) parameters were used for the children aged 60–83 months. The cutoffs recommended by the WHO, which were used in the study for screening of under-nutrition and over-nutrition are presented in Table 2.

**Table 2:** The WHO cutoffs for screening of under-nutrition and over-nutrition

Parameter	2006 WHO standards	2007 WHO reference
Underweight (weight-for-age)	<−2 z-scores	<−2 z-scores
Severe underweight (weight-for-age)	<−3 z-scores	<−3 z-scores
Stunting (length-height-for-age)	<−2 z-scores	<−2 z-scores
Severe stunting (length-height-for-age)	<−3 z-scores	<−3 z-scores
Wasting (weight-for-length-BMI-for-age^a^)	<−2 z-scores	<−2 z-scores
Severe wasting (weight-for-height)	<−3 z-scores	<−3 z-scores
Risk of overweight (weight-for-length/BMI-for-age^a^)	>+1 z-scores	n/a
Overweight (weight-for-length/BMI-for-age^a^)	>+2 z-scores	>+1 z-score
Obese (weight-for-length/BMI-for-age^a^)	>+3 z-scores	>+2 z-score
Severe obesity (BMI-for-age)	n/a	>+3 z-scores

*n/a* not applicable

^a^Weight-for-length from birth to 2 years; BMI-for-age > 2 years

Anthropometric software provided by the WHO was used for conversion of data into anthropometric indices according to both, standards and WHO references [16, 17]. Statistical analyses were carried out using statistical package SPSS version 17.0. The mean values of body mass and stature across sex and age groups as well as means and SD of anthropometric indicators (HAZ, WHZ, and BAZ) between public and private kindergartens were compared. Qualitative variables were tested with a *chi-square* test. The differences between groups were assessed with a *Student t test* for normally distributed variables, and a *Mann-Whitney test* was used for not normally distributed numerical variables. The level of significance adopted for the statistical test was 5%.

## Results

This investigation, which was conducted in preschool settings in Kosovo, sought to construct a profile of the nutritional status of preschool children attending kindergartens in Kosovo.

Results of anthropometric indicators presented in Table 3 show that mean z-scores for weight-for-age, height-for-age, weight-for-height, and BMI-for-age largely fell within 0.0 and 1.0. For weight-for-age, which reflects the proportion of body mass to chronological age, the categories having the lowest mean z-scores were boys aged 72–<84 months (−0.1±1.4), while the categories having the highest mean z-scores were boys aged 12–<24 months (0.8 ± 1.2) and also girls aged 12–<24 months. Statistical tests did not show significant differences (*p ˃ 0.05*) in WAZ between girls and boys.

**Table 3:** Mean and standard deviations of WAZ, HAZ, WHZ, and BMI scores by age and sex

Age groups (months)	Female		Male		Total		*p* value
	Mean	SD	Mean	SD	Mean	SD	
	Weight for age (z-scores)						
12–23	0.8	0.9	0.8	1.2	0.8	1.1	0.979
24–35	0.7	1.2	0.6	1	0.7	1.1	0.700
36–47	0.2	1	0.4	1	0.3	1	0.200
48–59	0.2	0.9	0.5	1.1	0.4	1.1	0.278
60–71	0.4	1.1	0.3	1.1	0.3	1.1	0.572
72–83	0.6	0.9	−0.1	1.4	0.1	1.3	0.196
	Height for age (z-scores)						
12–23	0.3	1.1	1.1	2.3	0.8	2	0.664
24–35	0.3	1.5	−0.1	1	0.1	1.3	0.191
36–47	0.1	1.1	0.2	1	0.1	1	0.524
48–59	0	0.9	0.2	1	0.1	1	0.418
60–71	0.2	1.3	0.1	1	0.1	1.1	0.640
72–83	0.4	1.1	−0.2	1.2	0	1.2	0.179
	Weight for height (z-scores)						
12–23	0.8	0.7	0.4	1.1	0.6	1	0.207
24–35	0.7	1.1	0.9	1.1	0.8	1.1	0.458
36–47	0.2	1.1	0.4	1	0.3	1.1	0.233
48–59	0.3	0.9	0.5	1.1	0.4	1	0.634
60–71	0.7	1.7	0.6	0.7	0.7	1.3	0.904
	BMI for age (z-scores)						
12–23	0.9	0.6	0.3	1.3	0.5	1.1	0.123
24–35	0.8	1.2	0.9	1.1	0.8	1.2	0.520
36–47	0.2	1.2	0.4	1	0.3	1.1	0.266
48–59	0.3	0.9	0.5	1.1	0.5	1	0.524
60–71	0.4	1	0.3	1.1	0.4	1	0.663
72–83	0.5	0.7	0.1	1.4	0.2	1.2	0.427

For height-for-age, which reflects achieved linear growth, the categories having the lowest mean z-scores were again boys aged 72–<84 months (−0.2 ± 1.2), while the categories having the highest mean z-scores were boys aged 12–<24 months (1.1 ± 2.3). No significant differences (*p ˃ 0.05*) were noted in HAZ between girls and boys. For BMI-for-age as a recommended method for screening of overweight and underweight children, the categories having the highest mean z-scores were girls aged 12–<24 months (0.9 ± 1.2) and also boys aged 24–<35 months. Gender differences were statistically tested also for WHZ and BMI, and no significant differences (*p ˃ 0.05*) were noted between boys and girls.

Results of the *weight-for-length/height* and BMI-for-age growth indicators are presented in Table 4. Weight-for-length/height growth indicator showed that 2.9% of 24–<36-month-old children were less than −2 z-scores, thus suffering from moderate acute malnutrition. The −2 z-score values were observed also in two other groups: 2.4% in the age group of 24–<36-month-old children and 2.3% in the group of 12–<24-month-old children. It is observed that 2.3% of 12–<24-month-old children and 0.8% of 36–<48-month-old children are less than −3 z-scores suffering from severe acute malnutrition. In terms of gender, the highest prevalence of moderate acute malnutrition was observed in boys of the 12–<24-month-old group (3.7%) and in girls of 24–<36-month-old group. On the other side, the severe acute malnutrition was identified in boys of 12–<24-month-old group (3.7%) and in girls 36–<48 months old.

**Table 4:** Weight-for-length/height and BMI for age indicators

	Number	% <−3SD	(95% CI)	% <−2SD	(95% CI)	% >+1SD	(95% CI)	% >+2SD	(95% CI)	% >+3SD	(95% CI)
		Weight-for-length/height indicator									
Combined (all ages)											
12–<24	44	2.3	(0.2%, 19.5%)	2.3	(0.2%, 19.5%)	34.1	(13.7%, 62.8%)	6.8	(2.4%, 17.8%)	0	(−, −)
24–<36	70	0	(−, −)	2.9	(0.3%, 23.2%)	44.3	(25%, 65.5%)	12.9	(3.1%, 40.1%)	4.3	(1.9%, 9.3%)
36–<48	124	0.8	(0.1%, 7.2%)	2.4	(0.8%, 6.7%)	22.6	(9.6%, 44.6%)	5.6	(1.4%, 19.8%)	0.8	(0%, 11.7%)
48–<60	121	0	(−, −)	0	(−, −)	25.6	(14.7%, 40.7%)	9.1	(3.8%, 20.3%)	0.8	(0.1%, 5.3%)
Boys (all ages)											
12–<24	27	3.7	(0.3%, 30.4%)	3.7	(0.3%, 30.4%)	29.6	(7.4%, 68.8%)	7.4	(1.9%, 24.8%)	0	(−, −)
24–<36	35	0	(−, −)	2.9	(0.2%, 28.9%)	51.4	(34.6%, 68%)	14.3	(5.6%, 31.7%)	2.9	(0.1%, 38%)
36–<48	58	0	(−, −)	1.7	(0.1%, 24%)	24.1	(11.2%, 44.4%)	8.6	(1%, 46.9%)	0	(−, −)
48–<60	73	0	(−, −)	0	(−, −)	26	(13.5%, 44.2%)	9.6	(3.9%, 21.8%)	1.4	(0.2%, 8.4%)
Girls (all ages)											
12–<24	17	0	(−, −)	0	(−, −)	41.2	(15.8%, 72.3%)	5.9	(0.3%, 58.5%)	0	(−, −)
24–<36	35	0	(−, −)	2.9	(0.1%, 49.9%)	37.1	(18.2%, 61.1%)	11.4	(1.1%, 59.6%)	5.7	(1.5%, 19.2%)
36–<48	66	1.5	(0.2%, 12.3%)	3	(0.6%, 14.5%)	21.2	(7.1%, 48.5%)	3	(1%, 8.9%)	1.5	(0.1%, 19.5%)
48–<60	48	0	(−, −)	0	(−, −)	25	(13.4%, 41.9%)	8.3	(2.8%, 22.5%)	0	(−, −)
		BMI for age indicator									
Combined (all ages)											
12–<24	44	2.3	(0.2%, 19.5%)	2.3	(0.2%, 19.5%)	34.1	(13.7%, 62.8%)	4.5	(0.4%, 34.5%)	0	(−, −)
24–<36	70	1.4	(0%, 31.9%)	2.9	(0.3%, 23.2%)	45.7	(23.2%, 70.1%)	12.9	(3.1%, 40.1%)	4.3	(1.9%, 9.3%)
36–<48	124	0.8	(0.1%, 7.2%)	2.4	(0.8%, 6.7%)	22.6	(9.6%, 44.6%)	5.6	(1.4%, 19.8%)	0.8	(0%, 11.7%)
48–<60	124	0	(−, −)	0	(−, −)	25	(13.8%, 41%)	10.5	(4.1%, 24.1%)	2.4	(0.6%, 10%)
60–<72	91	0	(−, −)	0	(−, −)	22	(9.3%, 43.5%)	6.6	(3.1%, 13.4%)	1.1	(0.1%, 17.5%)
72–<83	33	0	(−, −)	3	(0.2%, 33.6%)	21.2	(7.5%, 47.1%)	9.1	(4.5%, 17.6%)	3	(0.2%, 33.6%)
Boys (all ages)											
12–<24	27	3.7	(0.3%, 30.4%)	3.7	(0.3%, 30.4%)	29.6	(7.4%, 68.8%)	7.4	(0.6%, 50%)	0	(−, −)
24–<36	35	0	(−, −)	2.9	(0.2%, 28.9%)	51.4	(34.6%, 68%)	14.3	(5.6%, 31.7%)	2.9	(0.1%, 38%)
36–<48	58	0	(−, −)	1.7	(0.1%, 24%)	24.1	(11.2%, 44.4%)	8.6	(1%, 46.9%)	0	(−, −)
48–<60	75	0	(−, −)	0	(−, −)	26.7	(14%, 44.7%)	12	(3.8%, 31.9%)	4	(0.9%, 15.9%)
60–<72	46	0	(−, −)	0	(−, −)	21.7	(11%, 38.5%)	6.5	(2.2%, 17.5%)	2.2	(0.1%, 32.5%)
72–<83	23	0	(−, −)	4.3	(0.2%, 45.2%)	21.7	(7.9%, 47.4%)	13	(5.4%, 28.3%)	4.3	(0.2%, 45.2%)
Girls (all ages)											
12–<24	17	0	(−, −)	0	(−, −)	41.2	(15.8%, 72.3%)	0	(−, −)	0	(−, −)
24–<36	35	2.9	(0.1%, 49.9%)	2.9	(0.1%, 49.9%)	40	(15.3%, 71.1%)	11.4	(1.1%, 59.6%)	5.7	(1.5%, 19.2%)
36–<48	66	1.5	(0.2%, 12.3%)	3	(0.6%, 14.5%)	21.2	(7.1%, 48.5%)	3	(1%, 8.9%)	1.5	(0.1%, 19.5%)
48–<60	49	0	(−, −)	0	(−, −)	22.4	(10%, 42.9%)	8.2	(2.6%, 22.6%)	0	(−, −)
60–<72	45	0	(−, −)	0	(−, −)	22.2	(5.1%, 60.2%)	6.7	(2.3%, 17.6%)	0	(−, −)
72–<83	10	0	(−, −)	0	(−, −)	20	(3.4%, 63.7%)	0	(−, −)	0	(−, −)

**Fig. 1:**
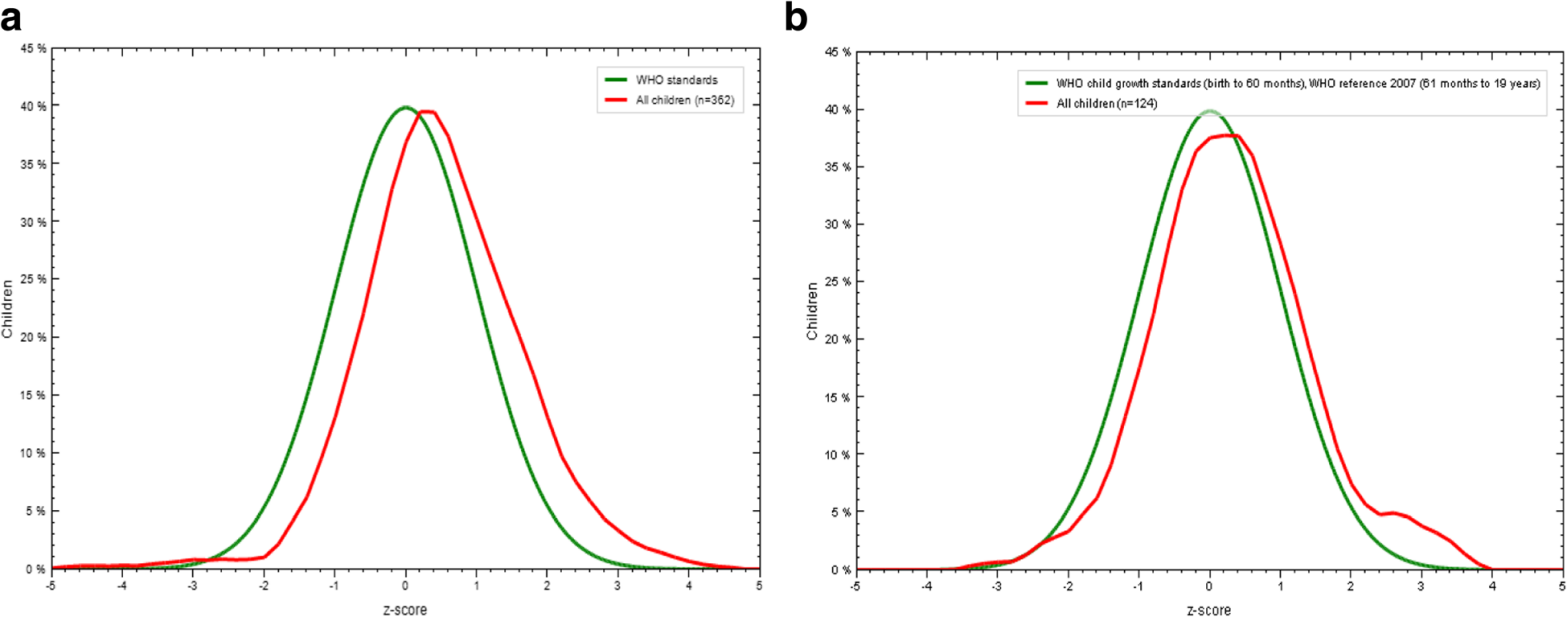
Distribution of BMI for age z-scores of Kosovo preschool children compared with the WHO standard distribution (**a**) children 12–59 months (**b**) children 60–83 months

BMI-for-age is used for screening for the risk of being overweight and for obesity in children aged 24–83 months. The percentage of obese children is 2.3%; furthermore, 8.9% are screened as overweight and 27.3% have a possible risk of being overweight. It is observed that 2.7% of boys and 1.4% of girls of the age group 24–83 months are obese, 10.9% of boys and 5.9% of girls were overweight, and 29.1% of boys and 25.2% of girls are at risk of being overweight. Distribution of BMI-for-age z-scores of children attending the kindergartens of Kosovo compared to the standard distribution of the *World Health Organization* is presented in Fig. 1.

## Discussion

This study showed that the percentage of underweight children among the 486 assessed children attending preschool institutions (12–83 months) is 0.7%, while 0.5% of the children are severely underweight. This percentage is lower than the average percentage from the study conducted in 2001 by the UNICEF [14], where the prevalence of underweight was 4%. The percentage of stunted children attending five kindergartens in Kosovo is 3%, and 0.7% are severely stunted. This measure of 3% is lower than the percentage from the study conducted in 2001 by the UNICEF, in which *low* height-for-age was found in 10% of the children aged 6–59 months. The weight-for-length/height shows that 1.9% of children (12–59 months) are less than −2 z-scores, thus suffering from moderate acute malnutrition or wasting and 0.8% of children are less than −3 z-scores suffering from severe acute malnutrition. This percentage is much lower than results from the UNICEF survey of July 1999 [13], in which the acute malnutrition was detected in 3.1% of the children from 0 to 5 years (including 1% severe) and chronic malnutrition was present in 10.7% of the children (including 3% severe).

Analyzing weight-for-age, length-height-for-age, and weight-for-length/height indicators, a reduction in underweight, stunting, and wasting of preschool-aged children from the year 1999 or 2001 could be seen in comparison to the time of this research. These results, which show a reduction in underweight when compared with results of research conducted by the UNICEF, may be not only due to improvement of living conditions in Kosovo after the war but also due to differences in sample characteristics of the present and previous studies.

## Conclusion

The results of BMI-for-age, which is used for screening of overweight and obesity in 24–83-month-aged children, showed that the prevalence of being overweight in children is evident. This investigation presents that the prevalence of being overweight in assessed children is a less firm; thus, there is a need for prevention and control programs. The obtained results from this study are in line with other international studies showing that children being overweight, and childhood obesity are increasing in less developed countries as well as in transitional societies [18–24].

The results of this study are also similar to those of other worldwide studies on the prevalence of overweight and obesity in preschool-aged children that report a growing trend in children being overweight [25–28].

These findings show that being underweight among preschool-aged children is low, but the prevalence of being overweight and obese in assessed children is evident. Based on the obtained results, we could conclude that there is a right time to prevent the malnutrition among preschool-aged children. The Kosovo Society and institutions should acknowledge the problem of malnutrition and its negative impact on the general health status of children.

The limitation of this study is that kindergartens in Kosovo have capacities to receive only a part of the preschool-aged children, so the results may not be indicative of the all preschool-aged children living in the country.

The next limitation of this study is that there are not enough background results on which to compare the present status of preschool children.

## Abbreviations

BAZ: BMI-for-age z-scores
BMI: Body mass index
EU: European Union
HAZ: Length-or height-for-age z-scores
SD: Standard deviation
SPSS: Statistical Package for the Social Sciences
UNICEF: United Nations Children’s Fund
WAZ: Weight-for-age z-scores
WHO: World Health Organization
WHZ: Weight-for-height z-scores)
